# Extra- and intranuclear heat perception and triggering mechanisms in plants

**DOI:** 10.3389/fpls.2023.1276649

**Published:** 2023-10-04

**Authors:** Xiaolong Yang, Hongling Guan, Yinghua Yang, Yiting Zhang, Wei Su, Shiwei Song, Houcheng Liu, Riyuan Chen, Yanwei Hao

**Affiliations:** College of Horticulture, South China Agricultural University, Guangzhou, China

**Keywords:** global warming, heat stress, thermosensor, GBPL3, phyB, liquid-liquid phase separation, transcriptional condensates

## Abstract

The escalating impact of global warming on crop yield and quality poses a significant threat to future food supplies. Breeding heat-resistant crop varieties holds promise, but necessitates a deeper understanding of the molecular mechanisms underlying plant heat tolerance. Recent studies have shed light on the initial events of heat perception in plants. In this review, we provide a comprehensive summary of the recent progress made in unraveling the mechanisms of heat perception and response in plants. Calcium ion (Ca^2+^), hydrogen peroxide (H_2_O_2_), and nitric oxide (NO) have emerged as key participants in heat perception. Furthermore, we discuss the potential roles of the NAC transcription factor NTL3, thermo-tolerance 3.1 (TT3.1), and Target of temperature 3 (TOT3) as thermosensors associated with the plasma membrane. Additionally, we explore the involvement of cytoplasmic HISTONE DEACETYLASE 9 (HDA9), mRNA encoding the phytochrome-interacting factor 7 (PIF7), and chloroplasts in mediating heat perception. This review also highlights the role of intranuclear transcriptional condensates formed by phytochrome B (phyB), EARLY FLOWERING 3 (ELF3), and guanylate-binding protein (GBP)-like GTPase 3 (GBPL3) in heat perception. Finally, we raise the unresolved questions in the field of heat perception that require further investigation in the future.

## 1 Introduction

**Figure 1:**
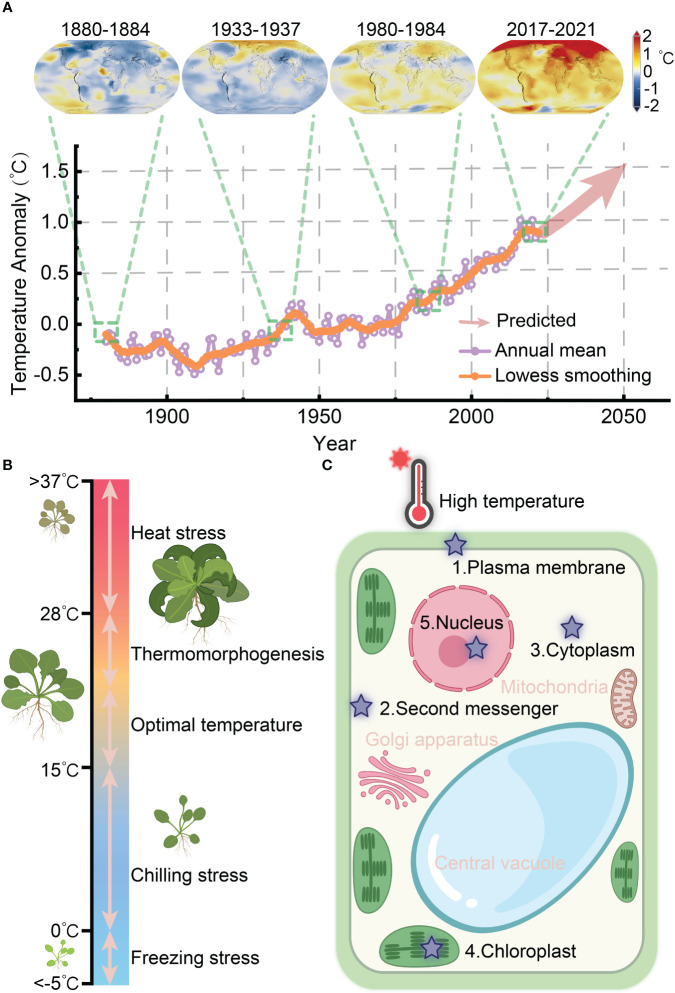
Heat perception and response of plants in coping with global warming. **(A)** The global surface temperature anomaly from 1880 to 2021 and expected warming trend up to 2050 (the data source: NASA/GISS https://climate.nasa.gov/vital-signs/global-temperature/). **(B)** Schematic representation of plant growth in response to temperature ranges, spanning from freezing stress to heat stress. **(C)** Potential heat perception sites within plant cells.

According to the recently released evaluation report from the Intergovernmental Panel on Climatic Change (IPCC) in 2023, global warming is accelerating and it is projected to rise by 1.5°C in the near term (2021–2040) due to the elevated levels of greenhouse gas emissions resulting from human activities (IPCC, 2023) (**Figure 1A**). The frequency of extreme heat-induced damage to crops has significantly risen, posing a critical threat to global food security. Furthermore, the global population is expected to reach 9.7-10 billion by 2050, up from the current population of 7.5 billion (Gupta et al., 2020). Enhancing plants’ ability to acclimate and endure high temperatures holds great promise as a solution to these challenges. Thermomorphogenesis and thermotolerance are the two primary responses employed by plants to cope with elevated temperatures (**Figure 1B**). As sessile organisms, plants rapidly response to heat by undergoing heat perception, signal transduction, and physiological changes to establish thermotolerance.

Heat perception serves as the initial event in the heat response process, decoding the initial stimulus to initiate specific cellular responses, known as the themosensory mechanism. However, the precise primary thermosensor(s) in plants have not been definitively identified. Essentially, a thermosensor would perceive elevated temperature by directly modifying its biochemical properties, transmit this information to downstream thermal responses, and ultimately generate physiological and morphological changes as outputs (Dai Vu et al., 2019; Kerbler and Wigge, 2023). Generally, heat can be sensed throughout a plant cell, and multiple thermosensing and overlapping downstream signaling pathways operate cocurrently. Proposed mechanisms for plant thermosensation include plasma membrane fluidity, photoreceptors, DNA/chromatin structures, alternative splicing, protein translation, protein stability, and phase separation (Kerbler and Wigge, 2023).

The quest for a bona fide thermosensor presents challenges due to technical limitations, genetic functional redundancy, and the complexity of phenotypic responses (Lamers et al., 2020). Nevertheless, significant progress has been made in recent years in characterizing components involved in heat perception, including phytochrome B (phyB), EARLY FLOWERING 3 (ELF3), thermo-tolerance 3.1 (TT3.1), and guanylate-binding protein (GBP)-like GTPase 3 (GBPL3) (Jung et al., 2016; Jung et al., 2020; Kim et al., 2022; Zhang et al., 2022). In this review, we specifically focus on the recent advances in understanding the heat perception mechanisms in plants, categorizing the initial process into extracellular and intracellular levels. This division is warranted because extracellular perception not only triggers a fast transcription-independent physiological response but also initiates a slower transcription-dependent response through the activation of signaling pathways. We particularly emphasize the involvement of second messengers, such as calcium ions (Ca^2+^), hydrogen peroxide (H_2_O_2_), and nitric oxide (NO), as well as plasma membrane-associated proteins, cytoplasmic and chloroplast components, and intranuclear transcriptional condensates that mediate heat perception and trigger response mechanisms in plants (**Figure 1C**).

## 2 Heat perception is mediated by second messenger

### 2.1 Ca^2+^ signal

As the most prominent second messenger, Ca^2+^ serves as a crucial link between extracellular abiotic and biotic stimuli and specific intracellular biological responses. Stress sensors recognize these stimuli and activate Ca^2+^–permeable cation channels, resulting in transient Ca^2+^ currents from the apoplastic space or intracellular vacuoles to the cytosol (Tian et al., 2020; Dindas et al., 2021). Various Ca^2+^-binding sensor proteins, including calcium dependent kinases (CDPKs), Calmodulin (CaM), CaM-like proteins (CMLs), and calcineurin B-like proteins (CBLs), decode the cytosolic Ca^2+^ signal and modulate downstream physiological responses (Luan, and Wang, 2021; Dong et al., 2022). Recent advances have shed light on early Ca^2+^-mediated signaling events that regulate pollen tube reception, low potassium responses, and immune response initiation (Jacob et al., 2021; Gao et al., 2022; Li et al., 2023a; Zhao et al., 2023). Notably, the combination of genetically encoded Ca^2+^ indicators (GECI) and forward genetics has facilitated the successful discovery of key stress sensors by screening mutants with altered cytosolic [Ca^2+^] (Yuan et al., 2014; Jiang et al., 2019; Wu et al., 2020; Grenzi et al., 2021; Jacob et al., 2021; Sun et al., 2021).

In plants, the cytosolic calcium concentration ([Ca^2+^]cyt) rapidly increases to initiate the earliest signaling events when exposed to low temperature conditions (Knight et al., 1991; Liu et al., 2021; Ding et al., 2022). This temperature-dependent transient burst of Ca^2+^ response may be attributed to either decreased plasma membrane fluidity or mediated through membrane-localized Ca^2+^ channels (Zhu, 2016; Gong et al., 2020). The plasma membrane protein CHILLING-TOLERANCE DIVERGENCE 1 (COLD1) has been identified as a potential cold sensor in rice. It is thought to interact with cold-responsive Ca^2+^ channels to stimulate apoplastic Ca^2+^ flux into the cytosol (Ma et al., 2015). Additionally, the mechanosensitive (MS) ion channels MID1-COMPLEMENTING ACTIVITY (MCA) proteins, namely MCA1 and MCA2, have been identified as responsive to cold stimuli and capable of inducing cytosolic Ca^2+^ influx (Mori et al., 2018; Yoshimura et al., 2021). The glutamate receptor channel-like Ca^2+^ channels protein GLR1.2, GLR1.3 and GLR3.4 are also sensitive to cold (Meyerhoff et al., 2005; Zheng et al., 2018). Cyclic nucleotide-gated calcium channels 9 (CNGC9) respond to cold treatment by triggering Ca^2+^ influx into the cytosol (Wang et al., 2021a). Ca^2+^-dependent phospholipid-binding proteins known as annexins also possess Ca^2+^ channel characteristic. ANN1, ANN3 and ANN5 have been described as positive regulators that mediate cold-induced calcium signaling in plants (Shen et al., 2017; Liu et al., 2021). These cold sensitive Ca^2+^ gating proteins may serve as potential sensory mechanisms in response to external cold stimulation, activating downstream signaling cascades.

**Figure 2:**
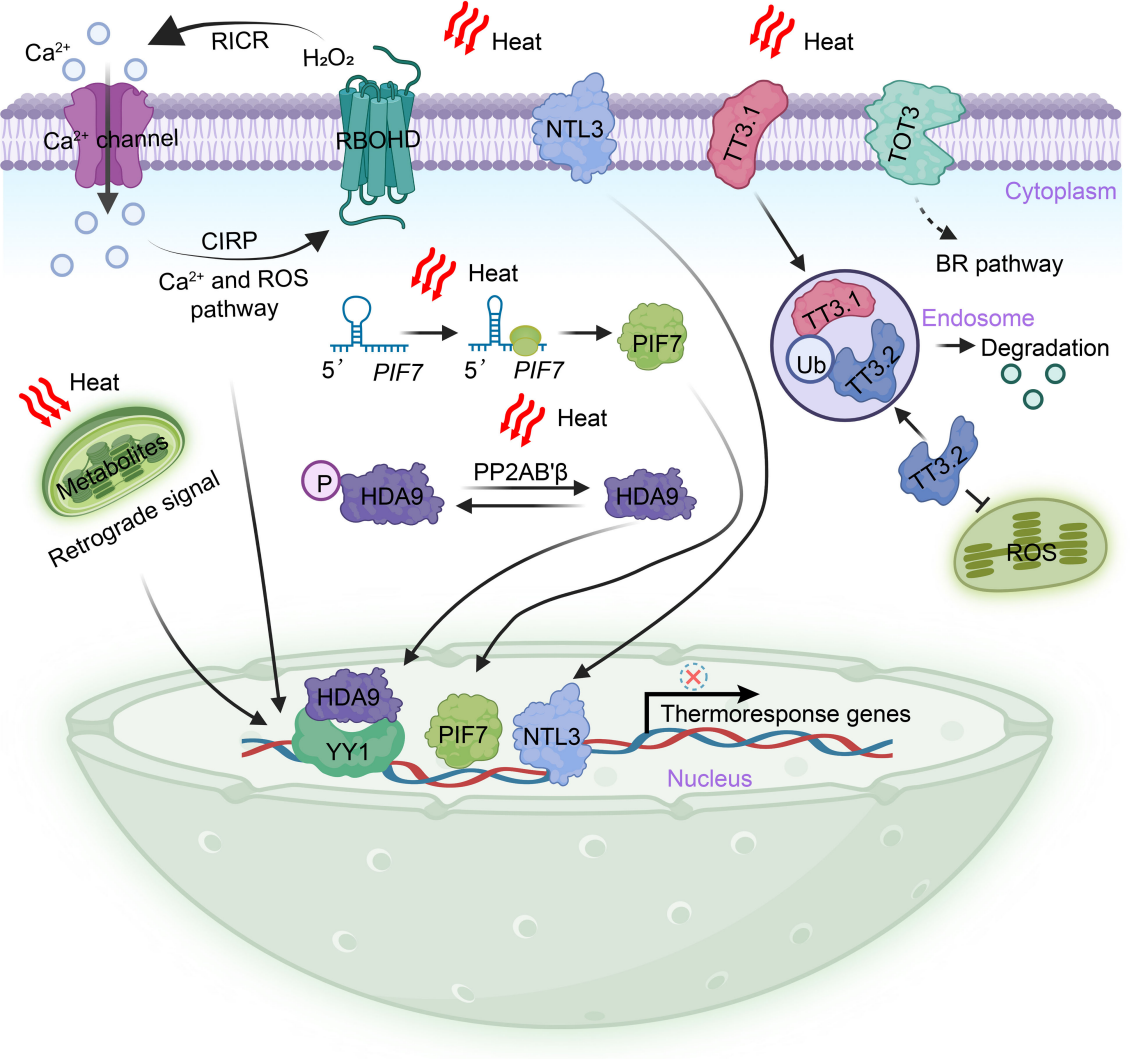
Extranuclear heat perception and triggering mechanisms in plants. The perception of heat in plants occurs outside the nucleus and involves various mechanisms. Heat stimuli are transmitted to the nucleus through a burst of heat-induced second messengers, including Ca^2+^ and H_2_O_2_. These second messengers play a crucial role in inducing the expression of thermoresponse genes. Additionally, regulatory circuits or homeostats can be formed by H_2_O_2_ and Ca^2+^, which provide feedback functions. During heat stress, specific cell surface-localized proteins, namely NTL3, TOT3, and TT3.1, mediate heat perception. These proteins translocate into the nucleus, where they induce the expression of heat response genes, activate the BR signal pathway, and maintain the integrity of chloroplasts. In the cytoplasm, the dephosphorylation of HDA9 by the PP2AB’β subunit of PROTEIN PHOSPHATASE 2A results in the stabilization and translocation of HDA9 into the nucleus. Once in the nucleus, HDA9 represses the expression of target genes through pathways that are both dependent and independent of the transcription factor YY1. The hairpin structure of *PIF7* mRNA is capable of perceiving heat and leads to an increased abundance of the PIF7 protein in the nucleus. This, in turn, activates the expression of thermoresponse genes. Heat stress also induces extensive metabolic remodeling, some of which can regulate gene expression in the nucleus through retrograde signaling. BR, brassinosteroids; CIRP, Ca^2+^-induced ROS-production; HDA9, HISTONE DEACETYLASE 9; NTL3, NAC transcription factor NTM1-like 3; P, phosphate; PP2AB′β, ′β regulatory subunit of PROTEIN PHOSPHATASE 2A; PIF7, Phytochrome interacting factor 7; RICR, ROS–induced Ca^2+^-release; RBOHD, respiratory burst oxidase homologue D; TT3.1, thermo-tolerance 3.1; TT3.2, thermo-tolerance 3.2; Ub, Ubiquitination; TOT3, TARGET OF TEMPERATURE 3; YY1, YIN YANG 1.

Similarly, a transient increase in [Ca^2+^]cyt following a heat stimulus serves as an early signaling event necessary for the positive regulation of thermotolerance. In mammals, the perception of high temperatures is mediated by the TRANSIENT RECEPTOR POTENTIAL (TRP) cationic channel family and the TWIK-RELATED POTASSIUM (TREK) channel family, which promote the influx of extracellular Ca^2+^ (Basbaum et al., 2009). In plants, exposure to heat activates the cyclic nucleotide-gated calcium channels CNGCb and CNGC2/4/6, resulting in their transient opening and subsequent entry of extracellular calcium, resulting in the induction of heat shock protein (HSP) gene expression and the acquisition of thermotolerance (Saidi et al., 2009; Finka et al., 2012; Gao et al., 2012). Notably, CNGC6 has been demonstrated to positively regulate the generation of H_2_O_2_ and NO by increasing [Ca^2+^]cyt upon heat exposure (Peng et al., 2019; Wang et al., 2021b). Additionally, CNGC14 and CNGC16 can enhance both chilling and heat tolerance in rice (Cui et al., 2020). While lacking genetic evidence, a study proposed that glutamate receptor-like (GLR) calcium channels, which mediate calcium signaling, may enhance heat tolerance and have the potential to function as thermosensors (Li et al., 2019). GLRs are known to facilitate systemic leaf–to–leaf wound signaling through the vasculature, and it is plausible that plants can also transmit systemic Ca^2+^ signals from a heated leaf to neighboring leaves (Mousavi et al., 2013; Nguyen et al., 2018; Simon et al., 2022; Yu et al., 2022). Furthermore, the Ca^2+^-dependent phospholipid-binding annexins ANN1 and ANN5 have been identified as Ca^2+^-permeable channels that respond to heat, with their transcriptional capacity being repressed by the R2R3-MYB transcription factor MYB30 (Qiao et al., 2015; Liao et al., 2017). These findings propose the hypothesis that calcium channels and/or their associated regulators present at the plasma membrane may function as thermosensors (**Figure 2**).

### 2.2 H_2_O_2_ and NO signal

Reactive oxygen species (ROS) and reactive nitrogen species (RNS) play pivotal roles as second messengers in the initial stages of the heat response. It remains critical to further explore how ROS signals are perceived and transduced into the nucleus during heat responses. In plants, H_2_O_2_ is generated by the plasma membrane-bound respiratory burst oxidase homologue D (RBOHD), which is sensitive to various abiotic stresses, including heat (Wang et al., 2014). Both H_2_O_2_ and Ca^2+^ can form regulatory circuits or homeostatic feedback mechanisms (Gilroy et al., 2014). The N terminus of RBOHD contains EF-hand motifs capable of binding to Ca^2+^, indicating that the production of H_2_O_2_ by RBOHD can be directly activated by changes of [Ca^2+^]cyt (Gilroy et al., 2014). Apoplastic H_2_O_2_ can induce the release of Ca^2+^ by directly modulating the activity of calcium channels or pumps. For instance, ANN Ca^2+^ channels and the vacuolar cation channel TWO PORE CHANNEL 1 (TPC1) are activated by ROS, leading to an increase in [Ca^2+^]cyt (Laohavisit et al., 2010; Evans et al., 2016). In *Arabidopsis*, H_2_O_2_-induced elevation of [Ca^2+^]cyt is mediated by HYDROGEN-PEROXIDE-INDUCED CALCIUM INCREASES 1 (HPCA1) (Wu et al., 2020). HPCA1 undergoes oxidation by H_2_O_2_, enhancing its kinase activity. This modified HPCA1 subsequently phosphorylates membrane-localized Ca^2+^ channels, facilitating calcium influx (Wu et al., 2020). ROS and Ca^2+^ have been demonstrated to propagate leaf-to-leaf long-distance signals rapidly in response to local stimuli, including heat, and are thus critical for systemic acquired acclimation (SAA) (Miller et al., 2009; Fichman et al., 2022) (**Figure 2**).

NO, a free radical of RNS, primarily functions as a signaling molecule that regulates plant adaptation to biotic and abiotic stresses (Fancy et al., 2017). Previous studies have demonstrated that heat exposure leads to an increase in NO levels, and both external application and genetic modification experiments support a positive role of NO in thermotolerance (Song et al., 2006; Peng et al., 2019; Rai et al., 2020; He et al., 2022). It is believed that NO acted downstream of Ca^2+^ and H_2_O_2_ signaling pathways to promote the accumulation of heat response proteins (Wang et al., 2014; Peng et al., 2019). A recent investigation uncovered that heat induces a burst of NO in the inflorescence apex, which then reacts with glutathione to form S-nitrosoglutathione (GSNO). GSNO rapidly travels through the vascular system to the root, where it acts as a signal to initiate whole-plant cellular heat responses through the S-nitrosylation of the GT-1 transcription factor. GT-1 subsequently binds to the promoter of the heat-inducible transcription factor *HsfA2*, thereby activating its expression and rapidly triggering downstream thermotolerance responses (He et al., 2022). These findings provide valuable insights into the precise mechanism by which NO functions in thermosensing. However, the regulatory relationships and mechanisms of interaction between NO, Ca^2+^, and H_2_O_2_ still require further elucidation.

## 3 Heat perception via cell surface–localized proteins

Changes in plasma membrane fluidity directly affected by heat can be sensed by membrane-localized proteins, although these potential heat transducers remain poorly understood. In addition to proteins involved in Ca^2+^ release and H_2_O_2_ production, recent studies have revealed that plasma membrane-embedded protein kinases, transcription factors, and E3 ligases can also sense heat and initiate downstream heat signaling responses (Liu et al., 2020; Vu et al., 2021; Zhang et al., 2022). NAM, ATAF, and CUC (NAC) transcription factors, known to be induced by abiotic stress and capable of enhancing stress tolerance, have emerged as key players in heat perception (Nakashima et al., 2012). For instance, the plasma membrane-localized NAC transcription factor NTL3 has been identified as a positive regulator of rice thermotolerance. A loss-of-function mutant of rice NTL3 displayed increased sensitivity to heat (Liu et al., 2020). In response to heat-induced changes in membrane fluidity, the plasma membrane-localized OsNTL3 is partially processed and translocate into the nucleus, where it activates the transcription of *OsbZIP74*, leading to the extensive expression of heat response genes (Liu et al., 2020). Such mechanisms promote the unfolded protein response of the endoplasmic reticulum (ER) and the detoxification of ROS (Liu et al., 2020) (**Figure 2**). These findings highlight the significance of NTL3 in facilitating communication among the plasma membrane, ER, and nucleus during heat perception. Additionally, the *Arabidopsis* NAC transcription factor NTL4, as well as rice NAC127 and NAC129, have also been shown to contribute positively to the heat response (Lee et al., 2014; Ren et al., 2021).

The accumulation of ROS due to photoinhibition can cause severe damage to chloroplast components under stress conditions, resulting in reduced crop yield and quality (Yang et al., 2019). Through quantitative trait locus (QTL) mapping, the first potential thermosensor in rice, TT3.1, was recently identified (Zhang et al., 2022). The TT3.1-TT3.2 genetic module is located at a single QTL locus and is crucial for linking plasma membrane heat perception to the maintenance of chloroplast quality, significantly reducing rice yield losses at high temperatures. TT3.1 is a RING-type E3 ligase localized to the plasma membrane, while TT3.2 binds to the chloroplast membrane and disrupts the core photoprotective apparatus under high temperatures. Overexpression of *TT3.1* or mutation of *TT3.2* significantly enhances thermotolerance capacity. Zhang et al. (2022) found that TT3.1 is released from the cell surface into the cytosol, where it recruits newly synthesized TT3.2 precursors into endosomes and polyubiquitinates TT3.2 for degradation in the vacuole. This study elucidates an important mechanism in which a plasma membrane-localized E3 ligase mediates heat perception and signal transduction, thereby preserving chloroplast integrity under high temperatures (**Figure 2**). Intriguingly, both NTL3 and TT3.1 are membrane-associated proteins that relocate from the plasma membrane to regulate downstream organelles in response to heat, indicating their pivotal roles as thermosensors in rice (Li & Liu, 2022).

Protein kinases play crucial roles in plant resistance and adaptation to unfavorable growing environments by sensing stress and transmitting signals. Various types of protein kinases, including CDPKs, mitogen-activated protein kinase (MAPK) cascades, receptor-like cytoplasmic kinases (RLCKs), and sucrose nonfermenting1 (SNF1)-related protein kinases (SnRKs), have been widely reported to regulate plant growth, development, and stress tolerance (Liang and Zhou, 2018; Chen et al., 2021; Li et al., 2022). These kinases influence the activity, localization, interaction, and stability of proteins through dynamic and reversible processes. For instance, the plasma membrane localized cold-responsive protein kinase (CRPK) can phosphorylate and transport 14-3-3 proteins to the nucleus, fine-tuning CBF signaling to regulate cold responses (Liu et al., 2017; Praat et al., 2021). However, our understanding of kinase-mediated phosphorylation events in the heat response remains limited. Therefore, the identification of plasma membrane-localized protein kinases that perceive heat and transmit perception signals to promote downstream physiological responses is an area of great interest. In rice, a plasma membrane-localized leucine-rich repeat receptor-like kinase (LRR-RLK) named Thermo-Sensitive Genic Male Sterile 10 (TMS10) was identified through a 60Co γ-ray radiation mutagenesis screen (Yu et al., 2017). Typically, an LRR-RLK protein consists of a transmembrane domain, a cytoplasmic kinase domain for signal transduction, and an extracellular domain for cell surface signal perception. Yu et al. (2017) demonstrated that both TMS10 and its redundant paralog, TMS10-Like (TMS10L), are important for controlling rice male fertility under lower temperatures, while only TMS10 is required for normal male fertility under heat conditions. A recent phosphoproteomics study identified the plasma membrane-localized mitogen-activated protein kinase kinase kinase kinase (MAP4K), known as TARGET OF TEMPERATURE 3 (TOT3), as an important and conserved thermoregulator in flowering plants (Vu et al., 2021) (**Figure 2**). TOT3 may perceive and transmit heat signals to control the activity of BRASSINAZOLE-RESISTANT 1 (BZR1), acting in parallel with the phyB-PIF4 pathway to regulate plant thermomorphogenesis (Vu et al., 2021). Nevertheless, further studies are required to fully understand how plasma membrane-localized protein kinases respond to heat.

## 4 Heat perception and signal transduction in cytoplasm

In addition to protein kinase-mediated phosphorylation, histone modifications play a crucial role in rapidly bridging heat signals to transcriptional programs (Han et al., 2022; Hou et al., 2022; Perrella et al., 2022; Bai et al., 2023). A recent study by Niu et al. (2022) demonstrated that HISTONE DEACETYLASE 9 (HDA9) acts as a novel and evolutionarily conserved cytoplasmic thermosensor responsible for transmitting heat signals to the nucleus. Previous research has shown that HDA9 participates in thermomorphogenesis by regulating H2A.Z-nucleosome dynamics, although the precise mechanism of the heat response remains unclear (Shen et al., 2019; Van Der Woude et al., 2019). Niu et al. (2022) identified that heat induces the interaction and dephosphorylation of HDA9 by the ′β regulatory subunit of PROTEIN PHOSPHATASE 2A (PP2AB’β) in the cytoplasm. This interaction leads to the stabilization of HDA9 and its rapid translocation into the nucleus. The accumulation of HDA9 in the nucleus is facilitated by the nuclear pore complex component HIGH EXPRESSION OF OSMOTICALLY RESPONSIVE GENE1 (HOS1) (Tasset et al., 2018; Niu et al., 2022). Within just 5 minutes of a temperature increase from 22°C to 37°C, the abundance of HDA9 in the nucleus significantly rises, underscoring its role as a typical signal transducer (Tasset et al., 2018; Niu et al., 2022). In the nucleus, HDA9 binds to and deacetylates the promoters of target genes, repressing their expression through pathways that are both dependent and independent of the transcription factor YIN YANG 1 (YY1). This intricate mechanism helps balance developmental and heat responses (Niu et al., 2022) (**Figure 2**). Given its fundamental properties, HDA9 has been emerged as a complex mediator in the heat response, serving as a potential cytoplasmic thermosensor in plants.

In the cytoplasm, mRNA translation efficiency is dynamically regulated by temperature fluctuations, primarily through the formation of thermolabile secondary structures in the 5′ untranslated region (UTR) (de Smit & Van Duin, 1990; Kortmann & Narberhaus, 2012). These secondary structures, resulting from intricate base pairing, expand the functional repertoire of mRNA beyond its DNA coding sequence (Vandivier et al., 2016). The formation of RNA secondary structures is highly responsive to abiotic stresses, and posttranscriptional covalent modifications may act as cytoplasmic thermosensors by modulating protein abundance (Assmann et al., 2023). Indeed, the concept of “RNA thermometers” has been previously proposed in bacteria and viruses and more recently observed in plants (Kortmann and Narberhaus, 2012; Chung et al., 2020; Meyer et al., 2020). For instance, the mRNA of phytochrome interacting factor 7 (PIF7) undergoes a temperature-dependent reversible change in secondary structure. Specifically, a hairpin structure consisting of 31 nucleotides in the 5′ UTR partially unfolds as the temperature increases, leading to enhanced translation efficiency of PIF7 mRNA and subsequent elevation of protein levels in the nucleus. Consequently, this activation triggers the transcription of key genes associated with thermomorphogenesis (Chung et al., 2020; Fiorucci et al., 2020) (**Figure 2**). PIF7 has been identified as the predominant transcriptional regulator of thermomorphogenesis and the shade avoidance syndrome in plants (Burko et al., 2022; Yang et al., 2023). These findings illustrate a thermosensory mechanism in which the hairpin structure of *PIF7* mRNA functions as a heat sensor in the cytoplasm, initiating a conformational change that transduces the thermal signal to the nucleus, ultimately leading to the induction of downstream transcriptional responses. Similar phenomena observed in *WRKY22* and *HSFA2* mRNA suggest that a conserved cytoplasmic thermosensory mechanism may operate widely in plants (Chung et al., 2020).

## 5 Heat perception in chloroplast

In addition to its primary role in energy and matter production, the chloroplast may also function as a critical center for heat perception and signal transduction. Leaves, with their high surface area/volume ratio, act as cooling mechanisms for plants. However, the intricate processes of chloroplast photosynthetic electron transport and metabolic reactions are highly sensitive to environmental fluctuations. Heat stress typically leads to the excessive accumulation of ROS in chloroplasts, which not only directly damages nearby microregions, particularly the photosynthetic apparatus and thylakoid membrane, but also initiates retrograde signaling to regulate cellular responses (Li, and Kim, 2021; Zahra et al., 2023) (**Figure 2**). Under heat stress conditions, chloroplasts undergo swelling, disorganization of thylakoid ultrastructure, an increase in the size and number of plastoglobules, and extensive metabolic remodeling (Djanaguiraman et al., 2018; Wang et al., 2018). Lipidome research has shown that heat stress significantly reduces the content of galactolipids and unsaturated phospholipids in tomato leaves, while increasing the content of triacylglycerols and decreasing lipid fatty acyl saturation in wheat leaf thylakoid membranes (Spicher et al., 2016; Djanaguiraman et al., 2018). This lipid composition remodeling plays a crucial role in protecting the photosynthetic apparatus by maintaining membrane stability. The proteins required for an intact chloroplast (approximately 2000–3000 proteins) are encoded by two spatially separate genomes: the chloroplast genomes, which encodes only around 100 proteins, and the nuclear genome, which encodes the majority of these proteins. Stressed chloroplasts undergo rapid protein turnover to restore homeostasis, necessitating the effective degradation of damaged proteins and the import of newly synthesized proteins. These processes heavily rely on chloroplast retrograde signaling (Woodson, 2022). Herein, we emphasize the crucial role of chloroplasts as both energy and matter-producing organelles, capable of acting as a potential center for heat signal sensing and transduction.

Retrograde signals serve as a communication language between the chloroplast and the nucleus, enabling the adjustment of cellular activities according to the extent of chloroplast damage (Chan et al., 2016; Sun and Guo, 2016). In plants, the absorption of excess light energy in photosystem (PSII) and photosystem I (PSI) leads to the production of ROS such as the superoxide anion (O_2_^−^), H_2_O_2_, hydroxyl radical (OH^−^), and highly reactive singlet oxygen (^1^O_2_) (Li and Kim, 2021). Among these, ^1^O_2_ is the most prominent ROS–associated retrograde signal and can modulate nuclear gene expression and stress response. Two spatially distinct sensors, EXECUTER proteins and β-carotene, are involved in mediating ^1^O_2_-triggered signaling (Dogra and Kim, 2020). For example, the shuttle protein EX1 detects ^1^O_2_ in the chloroplast and translocates to the nucleus, where it facilitates the expression of ^1^O_2_–responsive genes by interacting with WRKY transcription factors (Li et al., 2023b). Additionally, various chloroplast metabolites function as retrograde signals to induce the expression of stress-responsive nuclear genes. These metabolites include methylerythritol cyclodiphosphate (MEcPP), tetrapyrroles, oxidative products of β-carotene, and phosphonucleotide 3′-phosphoadenosine 5′-phosphate (PAP) (Chan et al., 2016). While it is hypothesized that chloroplasts act as heat-sensing organelles, the specific identification of thermosensor proteins, lipids, or other metabolites in the chloroplast requires further confirmation.

## 6 Intranuclear transcriptional condensates mediate heat perception

### 6.1 PhyB

**Figure 3:**
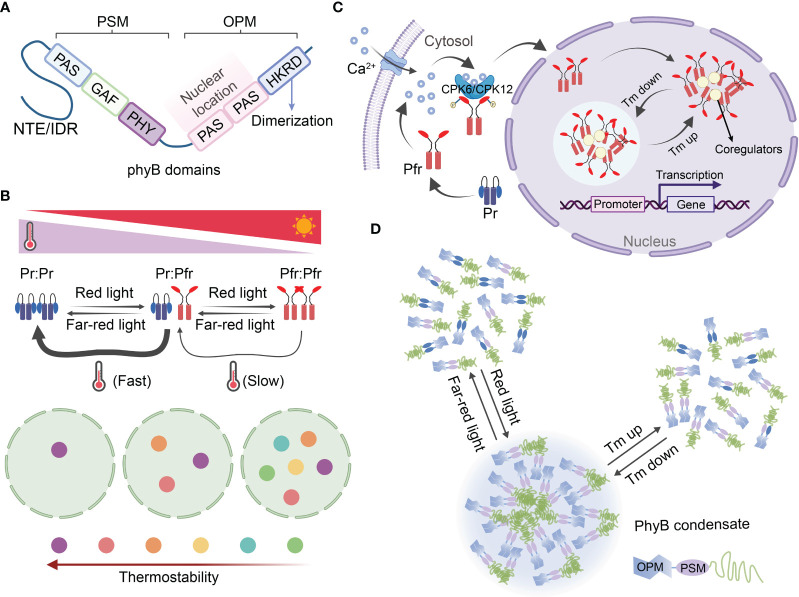
Photosensor phyB functions as themosensor in plants through reversible condensate formation. **(A)** Structural domains of phyB. The phyB protein can be divided into two structural domains: the C-terminal output module (OPM) and the N-terminal photosensory module (PSM). **(B)** The reversible photoconversion and thermal reversion of Pr-to-Pfr are key processes that contribute to the dynamics of phyB photobodies. Phytochromes are dimers that exhibit a highly dynamic nature due to photoconversion and thermal reversion of Pr-to-Pfr. Red light promotes the formation of the active dimer (Pfr-Pfr), while far-red light favors the inactive dimer (Pr-Pr). The active dimer pool is also influenced by thermal reversion, a two-step process that converts Pfr-Pfr back to the inactive Pr-Pr state. The kinetics of thermal reversion from Pfr : Pr to Pr : Pr are approximately two orders of magnitude faster than those from Pfr : Pfr to Pfr : Pr. The model depicting temperature-dependent phyB photobody (PB) dynamics is presented in the schematic below. The colored circles represent PBs with different thermostabilities, while the circle with a dotted line represents the nucleolus. Less thermostable PBs are only formed at lower temperatures, whereas increasing temperatures progressively induce the disassembly of individual PBs, which is dependent on the thermostability of phyB photobodies. Overall, this model provides insights into the reversible photoconversion and thermal reversion mechanisms of phyB, as well as the temperature-dependent dynamics of phyB photobodies. **(C)** A potential mechanism role of phyB import into the nucleus and photobody biogenesis through LLPS. The inactive Pr converting to active Pfr, and the activated Pfr was phosphorylated by CPK6/12 that induced by Ca^2+^, followed by rapidly import from the cytoplasm into the nucleus to form thermal reversion phyB photobodies. Red light can activate the Ca^2+^–CPK6/12 pathway via phyB, leading to the phosphorylation of the Pfr conformer and subsequent nuclear import. This phyB–Ca^2+^–CPK6/12–phyB regulatory loop, which operates in light signaling. Ultimately formed thermal reversion condensate through LLPS. Potential mechanism for the role of phyB in nuclear import and photobody biogenesis through liquid-liquid phase separation (LLPS). The inactive Pr form of phyB converts to the active Pfr form, which is then phosphorylated by CPK6/12 in response to Ca^2+^ signaling. Subsequently, the phosphorylated Pfr is rapidly imported from the cytoplasm into the nucleus, where it forms thermal reversion phyB photobodies. This phyB–Ca^2+^–CPK6/12–phyB regulatory loop operates in light signaling and ultimately leads to the formation of thermal reversion condensates through LLPS. **(D)** The light and temperature sensing mechanism of phyB involves the assembly and deformation of photobodies through LLPS. In its Pr form, phyB exists as a dimer and is unable to form a condensate. However, upon activation by red light, the conformational changes in the Pfr form allow for high-order assembly and drive condensate formation. Conversely, far-red light facilitates the disassembly of these condensates. Additionally, the multivalent interactions of phyB can be reversibly altered by changes in temperature, resulting in the assembly or disassembly of condensates. For a more comprehensive understanding, please refer to the study conducted by Chen et al. (2022). CPK6/CPK12, calcium-dependent protein kinase 6/calcium-dependent protein kinase 12; GAF, cGMP phosphodiesterase/adenylyl cyclase/FhlA domain; HKRD, histidine kinase-related domain; IDR, intrinsically disordered region; PAS, period/arnt/single-minded domain; OPM, output module; PHY, phytochrome-specific domain; PSM, photosensory module; Pr, biologically inactive red-light absorbing form of phyB; Pfr, biologically active far-red light-absorbing form of phyB; NTE, N-terminal extension; Tm, temperature.

The photoreceptor phyB is the first identified and the best-known thermosensor in plants, initiating both photomorphogenesis and thermomorphogenesis through specific signaling pathways (Jung et al., 2016; Legris et al., 2016). It was originally identified as a red/far-red light receptor, and has been the subject of extensive research in plants for many years (Butler et al., 1959). PhyB, consisting of a C-terminal output module (OPM) and a N-terminal photosensory module (PSM), functions in two forms: an inactive Pr form that absorbs red light and an active Pfr form that absorbs far-red light. Pr form and Pfr form could convert mutually according to the light quality. Upon exposure to red light, Pr converts to the active Pfr form, while far-red light irradiation reverts Pfr back to Pr (Cheng et al., 2021) (**Figure 3A**). In the absence of light, active Pfr can revert to the inactive Pr form, and this reversible conformational switch, known as thermal reversion, can also be induced by changes in temperature (Jung et al., 2016; Legris et al., 2016). When seedlings are subjected to high temperatures in the dark, the fraction of active phyB decreases, leading to an increase in the Pr/Pfr ratio. This higher Pr/Pfr ratio subsequently promotes hypocotyl elongation in seedlings (Jung et al., 2016; Legris et al., 2016) (**Figure 3B**).

PhyB, a dimeric chromoprotein, exhibits a highly dynamic nature due to the photoconversion and thermal reversion of Pr-to-Pfr. The kinetics of thermal reversion from Pfr : Pr to Pr : Pr are nearly two orders of magnitude faster than those from Pfr : Pfr to Pfr : Pr. Moreover, it is only the Pfr : Pfr pool in the nucleus that can initiate light and thermal responses (Klose et al., 2015; Legris et al., 2016; Klose et al., 2020). Biophysical analysis has demonstrated that phyB undergoes thermal reversion at a rate approximately ten times faster than other phy isoforms found in *Arabidopsis*, potato (*Solanum tuberosum*), and maize (*Zea mays*), suggesting that phyB is the primary thermosensor among the five isoforms in plants (Burgie et al., 2021) (**Figure 3B**). The rapid import of activated Pfr from the cytoplasm to the nucleus serves as an early gating step in information delivery (Chen & Chory, 2011; Van Buskirk et al., 2012). While the exact mechanism underlying phyB imported into the nucleus has remained unclear, a recent discovery by Zhao et al. (2023) revealed that red light can activate the Ca^2+^–CPK6/12 pathway via phyB, leading to the phosphorylation of the Pfr conformer and subsequent nuclear import. This phyB–Ca^2+^–CPK6/12–phyB regulatory loop, which operates in light signaling, likely participates in heat perception, as Ca^2+^ plays a ubiquitous role in plant responses to various stimuli (**Figure 3C**).

Phytochromes have the ability to homodimerize or heterodimerize with each other. As a result, the imported active Pfr forms small aggregates initially, which then assemble into subnuclear speckles known as photobodies (PBs). Conversely, the inactivated Pr is disassembled from photobodies. These subnuclear compartments have been referred to as speckles, foci, and nuclear bodies, but their biogenesis, components, and function have yet to be clearly confirmed. The size, number, and potentially the function of phyB photobodies are dynamically regulated by light and temperature. Under typical ambient temperatures, photobodies disappear, and phyB localizes to the numerous smaller subnuclear foci in the dark (or under far-red light) conditions, but not under red light. However, even under red light, the number of photobodies decreases at higher temperatures (Legris et al., 2016; Hahm et al., 2020). The intrinsically disordered region (IDR) of phyB contributes to the liquid-liquid phase separation (LLPS) process, resulting in the assembly of photobodies (Burgie et al., 2021; Pardi & Nusinow, 2021). Recently, a possible light and heat sensing mechanism of phyB through LLPS was proposed, suggesting that it plays a role in the assembly and deformation of photobodies (Chen et al., 2022). The intrinsically disordered N-terminal extension (NTE) and C terminus of phyB facilitate the spontaneous LLPS and oligomerization of phyB, allowing for its compartmentalization with transcription factors for signal transduction. The NTE acts as a biophysical modulator, directly influencing the temperature-dependent formation and dissolution of phyB droplets. Its conformational properties enable phyB to function both as a photoreceptor and a thermosensor (Viczián et al., 2020; Chen et al., 2022). Recent findings also support the notion that phyB photobodies can be regarded as “thermobodies” (TBs), given their characteristics related to heat perception (**Figure 3D**).

### 6.2 ELF3

**Figure 4:**
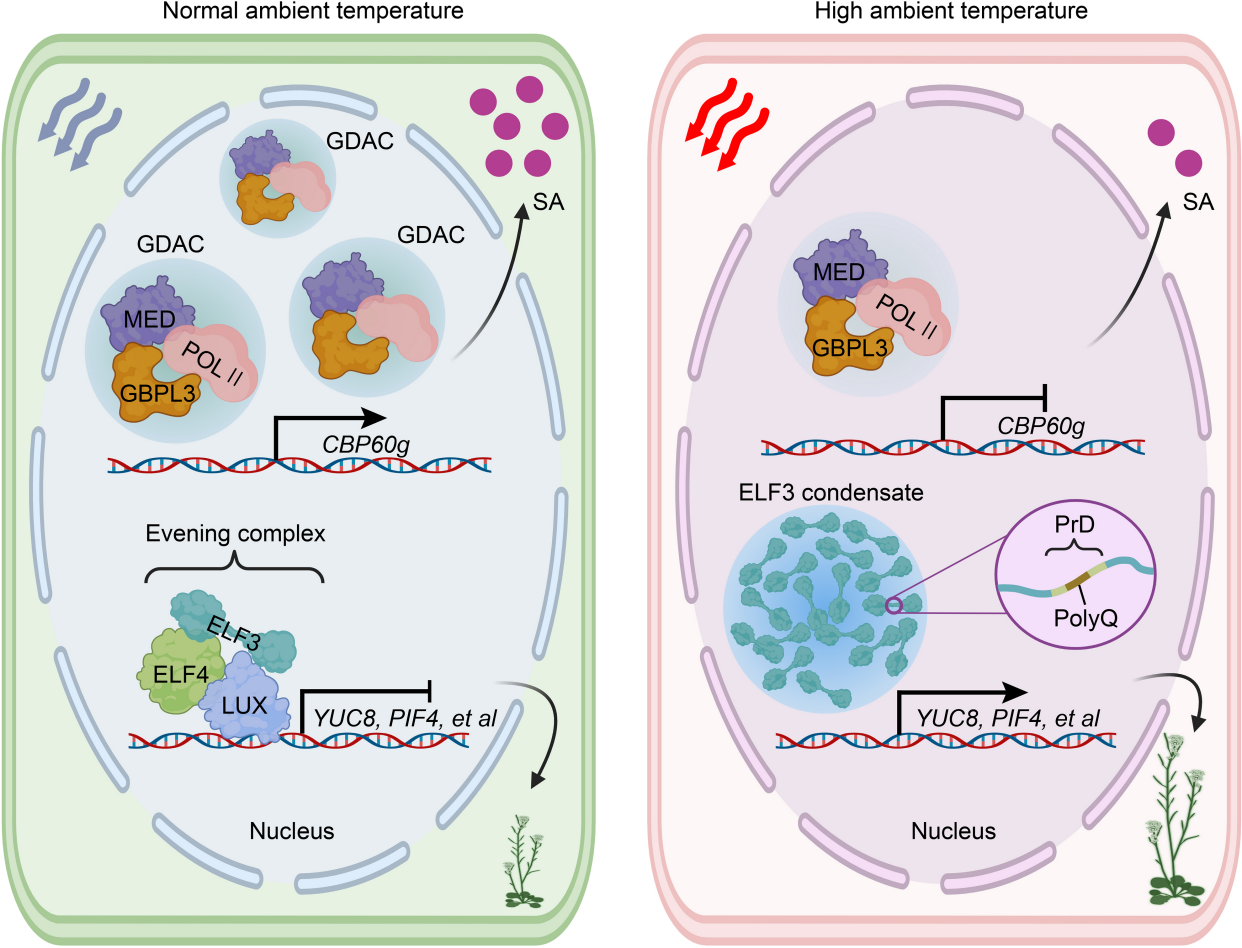
ELF3 and GBPL3 condensates play a crucial role in plants’ heat perception. Elevated ambient temperature inhibits the accumulation of GDAC, consequently repressing the transcription of CBP60g. This repression ultimately leads to the downregulation of SA biosynthesis and compromised immunity. Notably, ELF3 undergoes reversible condensate formation mediated by LLPS upon exposure to heat. Under normal ambient temperature conditions, ELF3 can form an evening complex by interacting with ELF4 and LUX, which subsequently represses the expression of YUC8, PIF4, and other relevant factors. However, the formation of ELF3 condensate induced by heat temporarily disrupts the functioning of the evening complex, consequently triggering thermalmorphogenesis. ELF3, EARLY FLOWERING 3; ELF4, EARLY FLOWERING 4; GDAC, GBPL defense-activated condensate; GBPL3, GUANYLATE BINDING PROTEIN-LIKE 3; LUX, LUX ARRYTHMO; MED, Mediator complex; POL II, RNA polymerase II; PIF4, phytochrome interacting factor 4; PolyQ, polyglutamine repeat; PrD, prion-like domain; YUC8, auxin biosynthetic protein YUCCA8; SA, salicylic acid.

The plasticity of plant morphology must dynamically respond to circadian rhythm (Silva et al., 2020; Xu et al., 2022). Recently, an important protein component of the circadian evening complex (EC), ELF3, was identified as a thermosensor in plant. Upon exposure to heat, ELF3 undergoes reversible liquid droplet formation driven by LLPS. These ELF3 droplets temporarily inhibit EC function, leading to the induction of PIF4 transcription (Jung et al., 2020). The C-terminal prion-like domain (PrD) of ELF3 plays a specific role in enabling the protein to aggregate into multiple speckles through LLPS in response to heat. Importantly, this process is reversible when temperatures return to normal (Jung et al., 2020; Lindsay et al., 2023). PrD regions are intrinsically disordered regions (IDRs) with low sequence complexity that are known to promote LLPS (Hennig et al., 2015; Dorone et al., 2021). Analysis of hypocotyl elongation and condensate formation has revealed a correlation between thermal responsiveness and the length of the polyglutamine repeat (PolyQ) within the PrD (Jung et al., 2020; Lindsay et al., 2023). A recent study further elucidated the potential biophysical basis for the thermal sensing mechanism of ELF3 involving the PrD region. It identified three processes contributing to ELF3 aggregation: the formation of temperature-sensitive helices adjacent to PolyQ tracts, increased solvent accessibility of expanded PolyQ tracts, and conformational changes that enhance the exposure of several aromatic residues at higher temperatures (Lindsay et al., 2023) (**Figure 4**). This study also proposed a potential mechanism for destabilizing the EC and facilitating the dissociation of ELF3.

It is noteworthy that there are multiple molecular mechanisms involved in the function of ELF3 in heat perception. For instance, N6-methyladenosine (m6A) RNA modification has been proposed as a facilitator of heat-induced formation of ELF3 condensates, independent of ELF3 phase separation (Lee et al., 2022). Recent studies have reported inconsistent results regarding the increased ELF3 nuclear condensates that disassemble the EC at warm temperatures. A genetic study demonstrated that ELF3 can act as a Zeitnehmer to transmit temperature cues to the central oscillator controlling thermoresponsive behaviors, independent of the EC (Anwer et al., 2020; Zhu et al., 2022). Another study found that ELF3 multimerization in the nucleus was reduced at warm temperatures, consistent with reports of ELF3 degradation through the ubiquitination degradation system (UPS) at warm temperatures (Ronald et al., 2021; Zhang et al., 2021a; Zhang et al., 2021b). These inconsistent findings may be attributed to variations in experimental conditions, molecular methods, or the length of the polyQ in the PrD of ELF3 among different *Arabidopsis* populations (Ronald et al., 2021). Moreover, the recent elucidation of the regulation of ELF3 protein stability through UPS revealed that the nuclear-localized proteins B-box 18 (BBX18) and BBX23 interact with ELF3 and the E3 ubiquitin ligase CONSTITUTIVE PHOTOMORPHOGENIC 1 (COP1) to promote ELF3 degradation at high temperatures in *Arabidopsis*. Additionally, the functionally redundant XB3 ORTHOLOG 1 IN ARABIDOPSIS THALIANA (XBAT31) and XBAT35 proteins perform a similar role (Ding et al., 2018; Zhang et al., 2021a; Zhang et al., 2021b).

### 6.3 GBPL3

The temperature sensor GUANYLATE BINDING PROTEIN-LIKE 3 (GBPL3), known for its ability to form GBPL defense-activated condensates (GDACs) through LLPS, has been identified recently. This unique property allows GBPL3 to connect external elevated temperatures to internal immunity by inducing a massive reprogramming of host disease-resistance gene expression (Huang et al., 2021; Kim et al., 2022; Tang et al., 2022). Under non-induced conditions, the higher-affinity chromatin-binding protein GBPL1 may compete directly with GBPL3 for chromatin binding. However, this competition can be reversed upon immune activation after phytopathogen infection. For instance, phytopathogen-induced salicylic acid (SA) can trigger the formation of GBPL3 condensates in the nucleus (Huang et al., 2021). In *Arabidopsis*, the nucleocytoplasmic relocation of GBPL3, along with C-terminal IDR- and N-terminal GTPase-dependent LLPS, plays a critical role in the formation of transcriptional condensates that activate defense gene expression and immunity. GBPL3 recruits the RNA polymerase II (POL II) apparatus and Mediator complex (MED) to form GDACs directly at the promoters of several major defense genes (Huang et al., 2021) (**Figure 4**).

Heat stress can directly impact various aspects of cellular physiology, making the plant immune system more vulnerable to pathogen attacks compared to normal growth temperatures (Janda et al., 2019; Saijo & Loo, 2020; Hua & Dong, 2022). SA, a versatile signaling molecule and essential defense phytohormone, plays a crucial role in activating plant immune gene expression and inducing resistance against diverse phytopathogens (van Butselaar and Van den, 2020; Peng et al., 2021; Jia et al., 2023). Higher temperatures or heatwaves have been shown to inhibit both basal and pathogen-induced levels of SA (Huot et al., 2017; Castroverde & Dina, 2021). Recently, Kim et al. (2022) demonstrated that elevated temperatures specifically suppress the recruitment of GBPL3, transcriptional coactivators, and RNA polymerase II (POL II) to form GDACs at the promoters of *CBP60g* and *SARD1*, which are crucial targets of SA suppression under high temperatures. Notably, this suppression occurs independently of the phyB and ELF3 thermosensors mentioned earlier. The GBPL3-dependent thermosensory mechanism offers a new framework that can be optimized to maintain immune capacity and ensure crop yields in the face of global warming (Dhar & Lee, 2022; Hua & Dong, 2022; Prasad et al., 2022).

## 7 Conclusions and perspectives

The escalating threat of higher temperatures owing to global warming poses a serious challenge to the survival of plants. Despite significant research efforts over the past few decades focusing on heat signal transduction and thermophysiological responses, there remains a challenging task of accurately defining thermosensors and conducting rigorous screening. Nonetheless, substantial progress has been made in unraveling heat sensing mechanisms at various levels within plant cells. Notably, the discovery in 2016 that the photoreceptor phyB also functions as a thermosensor has opened new avenues for research. Moreover, several potential thermosensors have been successfully identified in model plants such as *Arabidopsis thaliana* and rice. In this review, we have summarized the current understanding of heat perception and triggering mechanisms in plants, specifically categorizing them as extra- and intranuclear mechanisms. The insights discussed herein contribute to a deeper comprehension of plant thermotolerance mechanisms at the molecular level and hold promise for accelerating the production of new, more thermotolerant crop varieties by breeders.

Despite these breakthroughs in the field of plant heat perception, numerous unresolved questions still need further investigation. Several areas require attention and investigation in the near future are listed below:

a) Heat-induced alterations in physical forces may trigger cell wall integrity signaling, suggesting that the cell wall could also be serve as a site for heat perception.

b) We propose that the chloroplast may function as a heat perception organelle. However, identifying the specific sensory components within the chloroplast remains challenging.

c) The formation of LLPS–driven transcriptional condensates may represent a common mechanism underlying heat responses in plants. It is conceivable that new phase separation-mediated thermosensors may be discovered in the future. Further characterization is also necessary for different sensing mechanisms.

d) Multiple thermosensors are involved in mediating different physiological responses to meet the requirements of specific plant developmental stages. The coordination of these thermosensors in regulating downstream responses and determining the primary actor is yet to be determined.

e) Plant heat sensing and response should be examined from both temporal and spatial perspectives. How systemic heat signals are sensed and transmitted, as well as how thermal memory is stored, need to be unveiled.

f) Multiple stresses occur simultaneously or sequentially during plant growth, and it remains unclear how plants perceive and integrate heat signals with responses to other stresses.

## Author contributions

XY: Conceptualization, Visualization, Writing – original draft, Data curation, Funding acquisition, Investigation, Methodology, Software, Validation, Writing – review & editing. HG: Writing – review & editing, Formal Analysis, Methodology. YY: Validation, Writing – review & editing. YZ: Validation, Resources, Supervision, Writing – review & editing. WS: Resources, Project administration, Writing – review & editing. SS: Resources, Supervision, Writing – review & editing. HL: Resources, Supervision, Writing – review & editing. RC: Funding acquisition, Project administration, Supervision, Resources, Writing – review & editing. YH: Conceptualization, Funding acquisition, Project administration, Supervision, Validation, Writing – review & editing, Writing – original draft.
